# Validation of multiple myeloma risk stratification indices in routine clinical practice: Analysis of data from the Czech Myeloma Group Registry of Monoclonal Gammopathies

**DOI:** 10.1002/cam4.1620

**Published:** 2018-06-21

**Authors:** Jakub Radocha, Vladimír Maisnar, Luděk Pour, Ivan Špička, Jiři Minařík, Lenka Szeligová, Petr Pavlíček, Alexandra Jungová, Marta Krejčí, Tomáš Pika, Jan Straub, Lucie Brožová, Lukáš Stejskal, Adriana Heindorfer, Pavel Jindra, Petr Kessler, Peter Mikula, Michal Sýkora, Marek Wróbel, Jiří Jarkovský, Roman Hájek

**Affiliations:** ^1^ 4th Department of Medicine – Haematology Faculty of Medicine Charles University Hospital Hradec Králové Czech Republic; ^2^ Department of Internal Medicine, Hematology and Oncology University Hospital Brno Faculty of Medicine Masaryk University Brno Czech Republic; ^3^ 1st Medical Department – Clinical Department of Haematology of the First Faculty of Medicine General Teaching Hospital Charles University Prague Czech Republic; ^4^ Department of Hemato‐Oncology Faculty of Medicine and Dentistry University Hospital Olomouc Palacky University Olomouc Czech Republic; ^5^ Department of Haemato‐Oncology Faculty of Medicine University Hospital Ostrava University of Ostrava Ostrava Czech Republic; ^6^ Department of Internal Medicine and Hematology University Hospital Kralovske Vinohrady Prague Czech Republic; ^7^ Hematology and Oncology Department Charles University Hospital Pilsen Czech Republic; ^8^ Institute of Biostatistics and Analyses Faculty of Medicine Masaryk University Brno Czech Republic; ^9^ Department of Hematology Hospital Opava Opava Czech Republic; ^10^ Department of Hematology Hospital Liberec Liberec Czech Republic; ^11^ Department of Hematology and Transfusion Medicine Hospital Pelhrimov Pelhrimov Czech Republic; ^12^ Department of Clinical Haematology Hospital in Havirov Havirov Czech Republic; ^13^ Department of Clinical Hematology Hospital Ceske Budejovice Ceske Budejovice Czech Republic; ^14^ Department of Hematology Hospital Novy Jicin Novy Jicin Czech Republic

**Keywords:** Czech Myeloma Group Registry, monoclonal gammopathies, multiple myeloma, overall survival, real‐world, risk stratification

## Abstract

This study used data from the Czech Myeloma Group Registry of Monoclonal Gammopathies to validate the International Myeloma Working Group (IMWG) and revised International Staging System (R‐ISS) indices for risk stratification in patients with multiple myeloma (MM) in clinical practice. Patients were included if they had symptomatic MM, complete data allowing R‐ISS and IMWG staging (including cytogenetic information regarding t(4;14), t(14;16), and del(17p)), and key parameters for treatment evaluation. Median overall survival (OS) in included patients (n = 550) was 47.7 (95% CI: 39.5‐55.9) and 46.2 (95% CI: 38.9‐53.5) months from diagnosis and initiation of first‐line therapy, respectively. Patients categorized as higher vs lower risk had reduced survival; median OS from diagnosis was 35.4 (95% CI: 30.5‐40.3) vs 58.3 (95% CI: 53.8‐62.9) months in high‐risk vs other patients (IMWG;* P* = .001) and 34.1 (95% CI: 30.2‐38.0) vs 47.2 (95% CI: 43.4‐51.0) months in Stage III vs Stage II patients (R‐ISS;* P* < .001). In conclusion, IMWG and R‐ISS risk stratification indices are applicable to patients with MM in a real‐world setting.

## 1 INTRODUCTION

Multiple myeloma (MM) is a clinically heterogeneous disease, as evidenced by considerable variation in rates of response to treatment and overall survival (OS); indeed, OS in patients with MM has been shown to range from a few months to more than a decade.^1^ Much of the clinical heterogeneity of MM is thought to arise from multiple genomic events that result in tumor development and progression.^2^ These include genetic and epigenetic alterations, including point mutations, translocations (eg, t(4;14) and t(14;16)), deletions (eg, del(17p)),^3^ aberrant DNA and histone methylation,^4^ and/or abnormal microRNA expression.^5^

Several groups have worked to develop systems that use prognostic markers to stratify patients with MM into homogeneous survival subgroups.^1^, ^6^, ^7^, ^8^, ^9^ Risk stratification facilitates prognostication, allowing patients to be categorized as having lower risk (ie, longer OS) or higher risk (ie, shorter OS) disease.

In 2014, the International Myeloma Working Group (IMWG) recommended a new staging system based on (1) the criteria of the International Staging System (ISS) (which was first presented in 2005 and was based on high serum β_2_‐microglobulin and low serum albumin levels)^10^ and (2) cytogenetic information.^11^ With the IMWG system, patients are categorized as low risk (ISS Stage I/II and the absence of t(4;14), del(17p), and 1q21 and age <55 years), intermediate risk (neither low risk nor high risk), or high risk (ISS Stage II/III and t(4;14) or del(17p)). In 2015, a revised version of the ISS (R‐ISS) was presented,^1^ which incorporated chromosomal abnormalities detected by interphase fluorescent in situ hybridization (iFISH) and serum lactate dehydrogenase (LDH). With the R‐ISS, patients are categorized as Stage I (ISS Stage I and standard‐risk chromosomal abnormalities by iFISH and normal LDH), Stage II (neither Stage I nor Stage III), or Stage III (ISS Stage III and either high‐risk chromosomal abnormalities [del(17p) and/or t(4;14) and/or t(14;16)] by iFISH or high LDH). Despite the availability of these and other risk stratification indices, however, no single tool is uniformly used in patients with MM.

This study was undertaken to validate the IMWG and R‐ISS indices for risk stratification in a cohort of patients who were being treated in routine clinical practice, including in subgroups of patients who received new drugs and who underwent ASCT. Data for the study were derived from the Czech Myeloma Group Registry of Monoclonal Gammopathies (RMG), a large‐scale project that aims to monitor the diagnosis and treatment of monoclonal gammopathies in the Czech Republic and Slovakia.

## 2 SUBJECTS AND METHODS

This was a retrospective registry‐based analysis that used data derived from the RMG (https://rmg.healthregistry.org/). Details regarding the registry have been presented previously^12^, ^13^; in brief, the registry, which has become one of the flagship projects of the Czech Myeloma Group, was established in 2007 and retrospectively and prospectively collects data from patients with MM, monoclonal gammopathies of undetermined significance, amyloid light‐chain amyloidosis, and Waldenström's macroglobulinemia. In the Czech Republic, all patients with myeloma who are diagnosed undergo examination in one of the registry participating centers and the patient is registered at that time. Every year there is a recruitment of 200‐300 newly diagnosed patients which roughly corresponds to expected incidence of myeloma in Europe and patients who die early (after diagnosis) are captured as well. Parameters of interest captured by the registry include demographic data, disease characteristics, treatment choice(s), and response to treatment (including OS, TTP [time to progression], PFS [progression‐free survival], and time to next treatment) for each line of treatment and each treatment interval. Information regarding diagnosis, treatment response, and time‐to‐event endpoints is assessed according to current IMWG criteria. The registry is regularly monitored, and data are validated by an external monitor. Upon entering the RMG, all patients are required to sign an informed consent form for data collection; the consent forms are approved by the ethics committees of participating hospitals.

Patients were included in the current analysis if they had symptomatic MM, a complete dataset of parameters allowing R‐ISS and IMWG staging, and key parameters for treatment evaluation; parameters required for calculation of IMWG and R‐ISS scores included levels of albumin, β_2_‐microglobulin, and LDH and information regarding chromosomal abnormalities. As we were not able to obtain +1q data (these were not uniformly reported since the beginning of the registry), we simplified the analysis of IMWG score as high risk vs others. Cytogenetic data were obtained from multiple laboratories with various cutoffs for positivity reporting; depending on the threshold defined by each local laboratory, patients were considered positive for a translocation when it was present in a percentage ranging from 5% to 20% (with the most frequently reported cutoff value being 20%, as used in previous research).^1^ High LDH was defined as a serum level greater than the upper limit of normal. β_2_‐microglobulin testing and albumin testing were as per nationwide standardized methods.

Data were described by absolute and relative frequencies for categorical variables and by median (5th to 95th percentile) values for quantitative variables. For comparisons, the maximum likelihood Chi‐square test was used for categorical variables, while the Kruskal‐Wallis test was used for continuous variables. Using the Kaplan‐Meier method, TTP and OS were plotted. Kaplan‐Meier estimates were completed using the Greenwood confidence interval (CI), with a log‐rank test used to estimate the statistical significance of any differences between curves. A Cox proportional hazards model was performed to explore the univariate significance of risk factors. P values less than 0.05 were considered statistically significant (all tests were two‐sided). Analyses were performed using SPSS software (IBM Corp., released 2013, IBM SPSS Statistics for Windows, Version 23.0, Armonk, NY, USA) and software R version 3.2.3 (http://www.r-project.org).

## 3 RESULTS

Data from the RMG showed that the mean number of newly diagnosed cases of MM per year was 478; based on a population of approximately 10 million people, the annual incidence was estimated to be 4.8 per 100 000 individuals.

To be included in the current analysis, it was necessary that patients had valid data for chromosomal abnormalities. Patients were considered high risk if they were positive for at least one of the following genetic markers: t(4;14), t(14;16), or del(17p) and standard risk if they were negative in all the markers; 3460 patients in the RMG were excluded due to failure to meet this criterion. An additional 206 patients were excluded because of missing data relating to other parameters (eg, age, gender, and M‐protein type). As a result, data were analyzed in 555 MM patients included in the Czech Myeloma Group RMG; the majority of patients were included in the registry between May 2007 and April 2016 (with only 5 patients included before April 2007).

An overview of patient demographics, disease characteristics (ie, clinical and cytogenetic profiles), R‐ISS and IMWG categorization, and treatment patterns (including the receipt vs nonreceipt of new drugs [ie, PIs and IMiDs] and ASCT) is detailed in Table 1. Regarding IMWG risk stratification, 108 patients (19.5%) were classified as high risk and 447 patients (80.5%) were classified as nonhigh risk. For R‐ISS risk stratification, 97 (17.5%), 309 (55.7%), and 149 (26.8%) patients were classified as Stage I, Stage II, and Stage III, respectively. Of included patients, a minority (n = 181 [32.6%]) had undergone ASCT.

**Table 1:** Baseline and disease characteristics, R‐ISS and IMWG risk stratification status, and first‐line treatment (all patients)

N = 555^a^ ,^b^		n (%)	Median (5th‐95th percentile)
Sex			
Female		260 (46.8%)	—
Male		295 (53.2%)	
Age at diagnosis, y			
≤50		37 (6.7%)	66.0 (48.0‐80.0)
51‐60		119 (21.4%)	
61‐70		227 (40.9%)	
71‐80		145 (26.1%)	
>80		27 (4.9%)	
Length of follow‐up (mo)		—	22.2 (1.6‐60.5)
Durie‐Salmon stage (N = 553)			
I		67 (12.1%)	—
II		97 (17.5%)	
III		389 (70.3%)	
Durie‐Salmon substage			
A		407 (73.3%)	—
B		148 (26.7%)	
ECOG performance status (N = 545)			
0		94 (17.2%)	—
1		287 (52.7%)	
2		110 (20.2%)	
3‐4		54 (9.9%)	
Cytogenetic abnormalities			
t(4;14) (N = 550)			
Negative		486 (88.4%)	—
Positive		64 (11.6%)	
t(14;16) (N = 483)			
Negative		466 (96.5%)	—
Positive		17 (3.5%)	
del(17p) (N = 543)			
Negative		472 (86.9%)	‐
Positive		71 (13.1%)	
High risk	Presence of t(4;14)/t(14;16)/del(17p)	138 (24.9%)	—
Standard risk	Absence of t(4;14)/t(14;16)/del(17p)	417 (75.1%)	
LDH (ukat/L)^c^			
Normal	≤3.75	379 (68.3%)	3.2 (1.8‐6.3)
High	>3.75	176 (31.7%)	
M‐protein type			
IgG		346 (62.3%)	—
IgA		98 (17.7%)	
Light chain only		86 (15.5%)	
Other		25 (4.5%)	
Light‐chain type (N = 547)			
Kappa		339 (62.0%)	—
Lambda		198 (36.2%)	
Biclonal		10 (1.8%)	
Extramedullary mass (N = 543)			
No		481 (88.6%)	—
Yes		62 (11.4%)	
Bone marrow aspiration cytology (%) (N = 535)^c^			
≤20 (clonal plasma cells)		248 (46.4%)	—
>20 (clonal plasma cells)		287 (53.6%)	
Bone marrow histology (%) (N = 297)^c^			
≤20 (clonal plasma cells)		70 (23.6%)	—
>20 (clonal plasma cells)		227 (76.4%)	
Serum M‐protein quantity (g/L) (N = 529)		—	27.0 (0.0‐70.3)
Kappa/lambda ratio (N = 461)		—	7.6 (0.0‐1386.0)
Hemoglobin level (g/L)		—	103.0 (74.0‐143.0)
Thrombocyte count (10^9^/L)		—	213.0 (94.0‐375.0)
Calcium total level (mmol/L) (N = 554)		—	2.4 (2.0‐3.2)
Albumin level (g/L) (N = 529)		—	37.8 (23.9‐47.8)
Creatinine level (μmol/L)		—	100.0 (57.0‐513.0)
β_2_‐microglobulin (mg/L) (N = 549)		—	4.7 (1.9‐25.3)
CRP (mg/L) (N = 546)		—	4.9 (0.6‐52.9)
IMWG risk stratification			
High risk	ISS II/III and t(4;14)/del(17p)	108 (19.5%)	—
Other	Not high risk	447 (80.5%)	
R‐ISS risk stratification			
I	ISS Stage I and standard‐risk chromosomal abnormalities and normal LDH	97 (17.5%)	—
II	Not R‐ISS Stage I or Stage III	309 (55.7%)	
III	ISS Stage III and either high‐risk chromosomal abnormalities or high LDH	149 (26.8%)	
Treatment			
Bortezomib^b^		365 (65.8%)	—
Thalidomide^b^		174 (31.4%)	
Lenalidomide^b^		27 (4.9%)	
Carfilzomib^b^		7 (1.3%)	
PI (bortezomib/carfilzomib)^b^		372 (67.0%)	
IMiD (lenalidomide/thalidomide)^b^		201 (36.2%)	
Use of new drugs (IMiDs or PIs)			
Yes		505 (91.0%)	—
No		50 (9.0%)	
Use of ASCT (IMiDs or PIs)			
Yes		181 (32.6%)	—
No		374 (67.4%)	

ASCT, autologous stem cell transplantation; CRP, C‐reactive protein; del(17p), 17p deletion; ECOG, Eastern Cooperative Oncology Group; Ig, immunoglobulin; IMiD, immunomodulatory drug; IMWG, International Myeloma Working Group; ISS, International Staging System; LDH, lactate dehydrogenase; PI, proteasome inhibitor; R‐ISS; Revised International Staging System; t(4;14), 4;14 translocation; t(14;16), 14:16 translocation.

a Unless otherwise stated.

b Combinations of different drugs were possible.

c Conventional cutoffs were used.

The median follow‐up period was 22.2 months (5th‐95th percentile: 1.6‐60.5). Median OS was 47.7 months (95% CI: 39.5‐55.9) from the diagnosis of MM and 46.2 months (95% CI: 38.9‐53.5) from the initiation of first‐line therapy. Median PFS was 19.8 months (95% CI: 17.1‐22.5) from the initiation of first‐line therapy.

Table 2 summarizes IMWG risk stratification and demographic and disease characteristics of all patients at diagnosis. Table 3 summarizes R‐ISS risk stratification and demographic and disease characteristics of all patients at diagnosis. Regarding IMWG risk classification, statistically significantly higher proportion of Durie‐Salmon Stage III patients is present in the high‐risk group as well as patients with renal failure. Logically albumin, creatinine, and β_2_‐microglobulin levels differ significantly too (Table 2). Regarding R‐ISS risk stratification, there were significant differences across the 3 risk groups for multiple patient characteristics (Durie‐Salmon stage and substage, bone marrow aspiration cytology, and various clinical laboratory tests (eg, hemoglobin, thrombocyte count, albumin, creatinine, β_2_‐microglobulin, LDH, and CRP) (Table 3). For certain patient characteristics (eg, age, performance status, and M‐protein type), there was a significant association with R‐ISS risk stratification but not with IMWG risk stratification.

**Table 2:** Association between International Myeloma Working Group (IMWG) risk stratification and demographic and disease characteristics at diagnosis (all patients)

Characteristics at diagnosis^a^ (N = 555)^b^	IMWG risk stratification		*P* value^c^
	High risk (N = 108)	Other (N = 447)	
Age, y	67.0 (47.0‐77.0)	66.0 (49.0‐81.0)	.729
Sex			
Female	52 (48.1%)	208 (46.5%)	.763
Male	56 (51.9%)	239 (53.5%)	
First‐line therapy			
No new drugs	9 (8.3%)	41 (9.2%)	.783
New drugs (IMiD or PI)	99 (91.7%)	406 (90.8%)	
ASCT			
No	77 (71.3%)	297 (66.4%)	.330
Yes	31 (28.7%)	150 (33.6%)	
Durie‐Salmon stage (N = 553)			
I	3 (2.8%)	64 (14.4%)	<.001
II	17 (15.7%)	80 (18.0%)	
III	88 (81.5%)	301 (67.6%)	
Durie‐Salmon substage			
A	63 (58.3%)	344 (77.0%)	<.001
B	45 (41.7%)	103 (23.0%)	
Status Performance (N = 545)			
0	12 (11.4%)	82 (18.6%)	.179
1	54 (51.4%)	233 (53.0%)	
2	26 (24.8%)	84 (19.1%)	
3‐4	13 (12.4%)	41 (9.3%)	
M‐protein type			
IgG	60 (55.6%)	286 (64.0%)	.204
IgA	19 (17.6%)	79 (17.7%)	
Light chain only	24 (22.2%)	62 (13.9%)	
Other	5 (4.6%)	20 (4.5%)	
Light‐chain type (N = 547)			
Kappa	64 (59.8%)	275 (62.5%)	.572
Lambda	42 (39.3%)	156 (35.5%)	
Biclonal	1 (0.9%)	9 (2.0%)	
Extramedullary mass (N = 543)			
No	97 (90.7%)	384 (88.1%)	.442
Yes	10 (9.3%)	52 (11.9%)	
Bone marrow aspiration cytology (%) (N = 535)^d^			
≤20	33 (31.4%)	215 (50.0%)	.001
>20	72 (68.6%)	215 (50.0%)	
Bone marrow histology (%) (N = 297)^d^			
≤20	7 (14.3%)	63 (25.4%)	.080
>20	42 (85.7%)	185 (74.6%)	
Serum M‐protein quantity, g/L (N = 529)	37.4 (0.0‐80.2)	26.0 (0.0‐67.2)	.005
Kappa/lambda ratio (N = 461)	20.2 (0.0‐2111.1)	7.3 (0.0‐1221.8)	.525
Hemoglobin level, g/L	93.2 (68.0‐130.0)	107.0 (75.0‐144.0)	<.001
Thrombocyte count, 10^9^/L	183.0 (75.0‐372.0)	220.0 (109.0‐377.0)	<.001
Calcium total level, mmol/L (N = 554)	2.4 (2.0‐3.6)	2.4 (2.0‐3.1)	.141
Albumin level, g/L (N = 529)	34.0 (22.5‐45.5)	38.9 (25.0‐48.0)	<.001
Creatinine level, μmol/L	136.2 (68.0‐598.0)	93.0 (55.0‐490.0)	<.001
β_2_‐microglobulin, mg/L (N = 549)	7.5 (3.5‐40.0)	4.0 (1.8‐19.9)	<.001
LDH, ukat/L^d^	3.3 (1.7‐9.7)	3.1 (1.9‐5.9)	.083
CRP, mg/L (N = 546)	7.5 (1.0‐61.0)	4.2 (0.5‐48.0)	.001

ASCT, autologous stem cell transplantation; CRP, C‐reactive protein; Ig, immunoglobulin; IMiD, immunomodulatory drug; LDH, lactate dehydrogenase; PI, proteasome inhibitor.

a Categorical variables described by N (%); continuous variables described by median (5th‐95th percentile).

b Unless otherwise stated.

c Maximum likelihood Chi‐square test for categorical and Kruskal‐Wallis test for continuous variables.

d Conventional cutoffs were used.

**Table 3:** Association between Revised International Staging System (R‐ISS) risk classification and demographic and disease characteristics at diagnosis (all patients)

Characteristics at diagnosis^a^ (N = 555)^b^	R‐ISS risk classification			*P* value^c^
	Stage I (N = 97)	Stage II (N = 309)	Stage III (N = 149)	
Age, y	63.0 (48.0‐73.0)	67.0 (50.0‐81.0)	67.0 (44.0‐81.0)	<.001
Sex				
Female	50 (51.5%)	135 (43.7%)	75 (50.3%)	.243
Male	47 (48.5%)	174 (56.3%)	74 (49.7%)	
First‐line therapy				
No new drugs	9 (9.3%)	28 (9.1%)	13 (8.7%)	.988
New drugs (IMiD or PI)	88 (90.7%)	281 (90.9%)	136 (91.3%)	
ASCT				
No	47 (48.5%)	215 (69.6%)	112 (75.2%)	<.001
Yes	50 (51.5%)	94 (30.4%)	37 (24.8%)	
Durie‐Salmon stage (N = 553)				
I	25 (26.0%)	37 (12.0%)	5 (3.4%)	<.001
II	19 (19.8%)	51 (16.6%)	27 (18.1%)	
III	53 (54.2%)	219 (71.4%)	117 (78.5%)	
Durie‐Salmon substage				
A	96 (99.0%)	244 (79.0%)	67 (45.0%)	<.001
B	1 (1.0%)	65 (21.0%)	82 (55.0%)	
Performance status (N = 545)				
0	19 (20.0%)	61 (20.1%)	14 (9.5%)	<.001
1	63 (66.3%)	154 (50.8%)	70 (47.6%)	
2	12 (12.6%)	56 (18.5%)	42 (28.6%)	
3‐4	1 (1.1%)	32 (10.6%)	21 (14.3%)	
M‐protein type				
IgG	65 (67.0%)	192 (62.1%)	89 (59.7%)	.024
IgA	20 (20.6%)	60 (19.4%)	18 (12.1%)	
Light chain only	10 (10.3%)	41 (13.3%)	35 (23.5%)	
Other	2 (2.1%)	16 (5.2%)	7 (4.7%)	
Light‐chain type (N = 547)				
Kappa	64 (66.0%)	184 (60.5%)	91 (62.3%)	.871
Lambda	31 (32.0%)	114 (37.5%)	53 (36.3%)	
Biclonal	2 (2.1%)	6 (2.0%)	2 (1.4%)	
Extramedullary mass (N = 543)				
No	81 (87.1%)	271 (89.7%)	129 (87.2%)	.641
Yes	12 (12.9%)	31 (10.3%)	19 (12.8%)	
Bone marrow aspiration cytology, % (N = 535)^d^				
≤20	59 (62.8%)	140 (47.1%)	49 (34.0%)	<.001
>20	35 (37.2%)	157 (52.9%)	95 (66.0%)	
Bone marrow histology, % (N = 297)^d^				
≤20	17 (27.4%)	45 (26.8%)	8 (11.9%)	.027
>20	45 (72.6%)	123 (73.2%)	59 (88.1%)	
Serum M‐protein quantity, g/L (N = 529)	22.7 (1.0‐42.9)	27.6 (0.0‐74.1)	34.9 (0.0‐79.6)	.009
Kappa/lambda ratio (N = 461)	5.8 (0.0‐409.3)	6.7 (0.0‐1386.0)	30.7 (0.0‐2462.4)	.125
Hemoglobin level, g/L	122.0 (94.0‐152.0)	103.0 (74.0‐143.0)	92.0 (67.0‐125.0)	<.001
Thrombocyte count, 10^9^/L	230.0 (120.0‐378.0)	217.0 (106.0‐381.0)	183.0 (82.0‐364.0)	<.001
Calcium total level, mmol/L (N = 554)	2.4 (2.2‐2.7)	2.3 (2.0‐3.1)	2.4 (2.0‐3.8)	.028
Albumin level, g/L (N = 529)	41.2 (36.0‐49.9)	37.0 (23.5‐47.0)	34.5 (22.6‐46.0)	<.001
Creatinine level, μmol/L	75.0 (50.0‐123.0)	97.0 (55.0‐439.0)	178.0 (79.0‐726.0)	<.001
β_2_‐microglobulin, mg/L (N = 549)	2.6 (1.5‐3.4)	4.3 (1.9‐18.4)	9.0 (5.8‐39.7)	<.001
LDH, ukat/L^d^	2.8 (1.9‐3.6)	3.1 (1.8‐5.4)	4.2 (1.9‐9.7)	<.001
CRP, mg/L (N = 546)	2.9 (0.2‐20.0)	4.0 (0.7‐60.0)	9.0 (1.0‐60.6)	<.001

ASCT, autologous stem cell transplantation; CRP, C‐reactive protein; Ig, immunoglobulin; IMiD, immunomodulatory drug; LDH, lactate dehydrogenase; PI, proteasome inhibitor.

a Categorical variables described by N (%); continuous variables described by median (5th‐95th percentile).

b Unless otherwise stated.

c Maximum likelihood Chi‐square test for categorical and Kruskal‐Wallis test for continuous variables.

d Conventional cutoffs were used.

**Figure 1:**
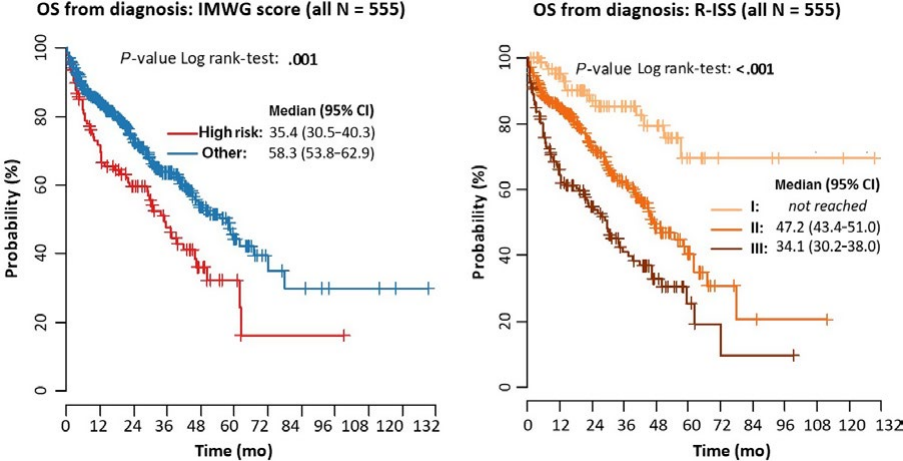
Overall survival (OS) from diagnosis according to International Myeloma Working Group (IMWG) and Revised International Staging System (R‐ISS) risk stratification (all patients [N = 555])

An analysis of the data showed that patients who were categorized as higher risk with both risk stratification indices had reduced survival. The median OS from diagnosis in patients who were categorized as high risk according to the IMWG criteria was 35.4 months (95% CI: 30.5‐40.3), compared with 58.3 months (95% CI: 53.8‐62.9) for all other patients (*P* = .001; Figure 1). Median OS from diagnosis in patients who were categorized as R‐ISS Stage III was 34.1 months (95% CI: 30.2‐38.0), compared with 47.2 months (95% CI: 43.4‐51.0) for patients categorized as Stage II (*P* < .001). Median OS from diagnosis for patients categorized as R‐ISS Stage I was not reached (Figure 1). An analysis of OS and PFS for both of the risk stratification systems is shown in Table 4. The data showed a statistically significant survival disadvantage for higher vs lower risk patients for all comparisons.

**Table 4:** Overall survival (OS) and progression‐free survival (PFS) according to International Myeloma Working Group (IMWG) and Revised International Staging System (R‐ISS) risk stratification indices (all patients [N = 555])

		OS from diagnosis	OS from treatment initiation	PFS
IMWG classification: High risk vs other	HR (95% CI)	1.69 (1.25‐2.29)	1.62 (1.19‐2.19)	1.45 (1.13‐1.87)
	*P* value	.001	.002	.004
R‐ISS Stage: II vs Stage I	HR (95% CI)	2.84 (1.66‐4.87)	2.67 (1.55‐4.57)	1.90 (1.34‐2.68)
	*P* value	<.001	<.001	<.001
R‐ISS Stage: Stage III vs Stage I	HR (95% CI)	5.20 (2.99‐9.03)	4.72 (2.72‐8.20)	2.41 (1.66‐3.48)
	*P* value	<.001	<.001	<.001

CI, confidence interval; HR, hazard ratio.

An analysis of OS and PFS according to ISS Stage, cytogenetic factors and LDH is shown in Table 5. The data showed a statistically significant survival disadvantage for the presence vs the absence of t(4;14) and the presence of high‐risk vs standard‐risk chromosomal abnormalities. The same was true for the presence of Stage II/III vs Stage I disease according to ISS criteria and for a per‐unit increase in LDH. There was a statistically significant survival disadvantage (PFS only) associated with the presence vs the absence of del(17p). In contrast, a statistically significant survival disadvantage (OS and PFS) was not found for the presence vs the absence of t(14;16) even though it has been described as a high‐risk chromosomal abnormality.

**Table 5:** Overall survival (OS) and progression‐free survival (PFS) according to cytogenetic abnormalities, International Staging System (ISS) Stage, and lactate dehydrogenase (LDH) (all patients [N = 555])

Risk factor: Risk vs reference category	OS from diagnosis		OS from treatment initiation	PFS
t(4;14): Positive vs negative	HR (95% CI)	1.55 (1.09‐2.22)	1.48 (1.04‐2.12)	1.42 (1.05‐1.91)
	*P* value	<.001	.030	.021
t(14;16): Positive vs negative	HR (95% CI)	0.46 (0.15‐1.45)	0.53 (0.17‐1.67)	0.94 (0.49‐1.84)
	*P* value	.185	.280	.866
del(17p): Positive vs negative	HR (95% CI)	1.42 (0.97‐2.07)	1.38 (0.94‐2.01)	1.45 (1.07‐1.98)
	*P* value	.070	.097	.017
Chromosomal abnormalities: High vs standard risk	HR (95% CI)	1.45 (1.08‐1.94)	1.42 (1.06‐1.91)	1.40 (1.10‐1.77)
	*P* value	.014	.018	.006
ISS: Stage II vs Stage I	HR (95% CI)	2.73 (1.70‐4.40)	2.53 (1.57‐4.07)	1.65 (1.21‐2.25)
	*P* value	<.001	<.001	.002
ISS: Stage III vs Stage I	HR (95% CI)	4.97 (3.237.64)	4.52 (2.94‐6.95)	2.18 (1.64‐2.89)
	*P* value	<.001	<.001	<.001
LDH (ukat/L): High vs normal	HR (95% CI)	1.19 (0.90‐1.59)	1.18 (0.89‐1.57)	1.11 (0.88‐1.40)
	*P* value	.222	.255	.373
LDH (ukat/L): Unit increase	HR (95% CI)	1.09 (1.05‐1.14)	1.09 (1.04‐1.14)	1.05 (1.01‐1.09)
	*P* value	<.001	<.001	.029

CI, confidence interval; HR, hazard ratio.

**Figure 2:**
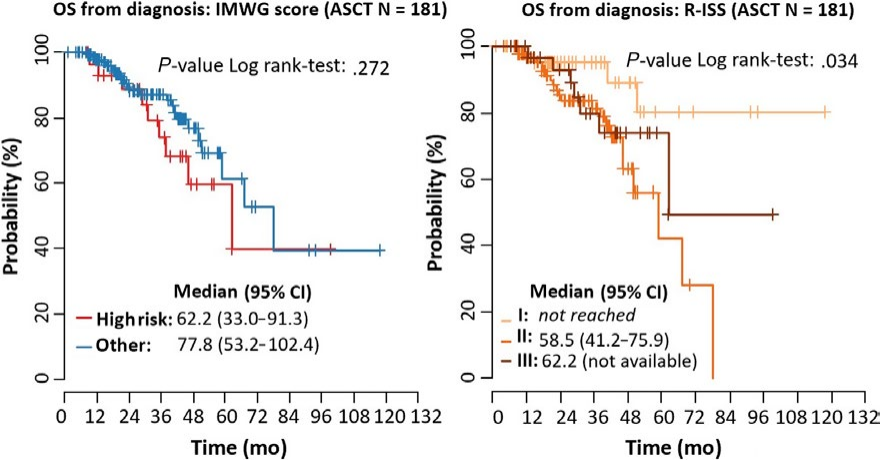
Overall survival (OS) from diagnosis and time to progression (TTP) for IMWG and R‐ISS stages (patients with ASCT; N = 181)

**Figure 3:**
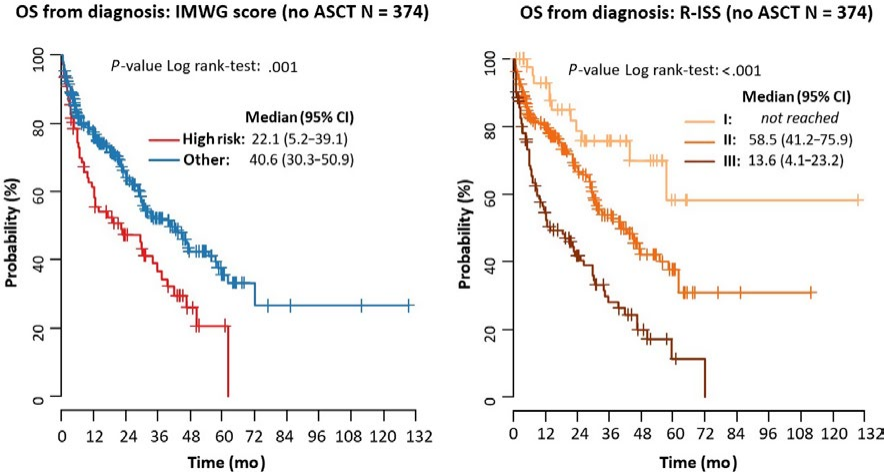
Overall survival (OS) from diagnosis and time to progression (TTP) for IMWG and R‐ISS stages (patients without ASCT; N = 374)

Regarding the IMWG criteria, median OS from diagnosis in patients who were categorized as high risk and who underwent ASCT was 62.2 months (95% CI: 33.0‐91.3); this was compared with 22.1 months (95% CI: 5.2‐39.1) for high‐risk patients who did not undergo ASCT. Corresponding values in non‐high‐risk patients were 77.8 months (95% CI: 53.2‐102.4) and 40.6 months (95% CI: 30.3‐50.9), respectively. Regarding the R‐ISS criteria, median OS from diagnosis in patients who were categorized as Stage III and who underwent ASCT was 62.2 months (95% CI: not available), compared with 13.6 months (95% CI: 4.1‐23.2) in Stage III patients who did not undergo ASCT. Corresponding values were 58.5 months (95% CI: 41.2‐75.9) and 40.6 months (95% CI: 29.5‐51.7), respectively, in Stage II patients. Median OS was not reached in Stage I patients. Corresponding survival curves are shown in Figures 2 and 3. An analysis of OS and PFS within each of the risk stratification systems according to treatment type (ie, no ASCT vs ASCT) is shown in Table 6. There was a survival disadvantage for higher vs lower risk patients for all comparisons. Results were uniformly statistically significant in the group of patients who did not undergo ASCT.

**Table 6:** Overall survival (OS) and progression‐free survival (PFS) according to International Myeloma Working Group (IMWG) and Revised International Staging System (R‐ISS) risk stratification indices and receipt vs nonreceipt of autologous stem cell transplantation (ASCT)

	Risk factor: risk vs reference category		OS from diagnosis	OS from treatment initiation	PFS
Patients without ASCT (N = 374)	IMWG score: High risk vs other	HR (95% CI)	1.72 (1.23‐2.40)	1.65 (1.18‐2.30)	1.37 (1.03‐1.83)
		*P* value	.001	.003	.032
	R‐ISS: Stage II vs Stage I	HR (95% CI)	2.18 (1.17‐4.08)	2.04 (1.09‐3.81)	1.59 (1.04‐2.41)
		*P* value	.015	.026	.031
	R‐ISS: Stage III vs Stage I	HR (95% CI)	4.69 (2.48‐8.84)	4.20 (2.23‐7.92)	2.16 (1.39‐3.36)
		*P* value	<.001	<.001	.001
Patients with ASCT (N = 181)	IMWG score: High risk vs other	HR (95% CI)	1.53 (0.71‐3.31)	1.44 (0.67‐3.11)	1.69 (1.00‐2.86)
		*P* value	.275	.350	.052
	R‐ISS: Stage II vs Stage I	HR (95% CI)	3.81 (1.29‐11.24)	3.52 (1.19‐10.42)	1.86 (0.98‐3.54)
		*P* value	.015	.023	.057
	R‐ISS: Stage III vs Stage I	HR (95% CI)	2.55 (0.74‐8.78)	2.28 (0.66‐7.84)	1.54 (0.73‐3.27)
		*P* value	.136	.192	.257

CI, confidence interval; HR, hazard ratio.

## 4 DISCUSSION

It is important that any risk stratification scheme is suitably validated. As part of its development, the original ISS was tested and found to be effective in patients from different geographic regions (North America, Europe, and Asia), patients of different ages (<65 and ≥65 years), patients receiving different treatments (standard therapy or autologous stem cell transplantation [ASCT]), and patients at different study sites (single sites and cooperative groups).^10^ In the same way, the R‐ISS was tested in patients of different ages (≤65 and >65 years) and in patients receiving different treatments (ASCT, proteasome inhibitors [PIs], or immunomodulatory drugs [IMiDs]).^1^ Although the ISS risk stratification tool has been evaluated outside of a clinical trial framework,^14^ the majority of patients (69.1%) on which the system was based were participating in a clinical trial^10^; similarly, the R‐ISS was based wholly on patients enrolled in experimental trials.^1^ This is relevant because patients in cancer trials may not be representative of the overall patient population and derivation of risk stratification criteria based on a highly selected set of patients may limit their applicability in the real world. As a result, testing of risk stratification criteria in population‐based studies is important. Validation in such studies is also important to determine the day‐to‐day practicality of conducting the tests that form the risk stratification criteria.

The current analysis was undertaken to validate the IMWG and R‐ISS indices for risk stratification in patients with MM in a real‐world setting. Using data from the Czech Myeloma Group RMG, it was shown that the prognostic value of the IMWG and R‐ISS indices for risk stratification is applicable to patients treated in routine clinical practice. These results extend the findings of studies conducted in patients who were, for the most part, participating in clinical trials.^1^, ^6^ Together, this study, and others of a similar nature,^15^, ^16^ indicate that the IMWG and R‐ISS indices for risk stratification are applicable to a broad spectrum of patients with MM.

### 4.1 Impact of LDH and cytogenetic abnormalities

The impact of LDH and cytogenetic changes in patients with MM has been well documented. Within the current cohort of patients, 31.7% had elevated LDH (ie, levels above the upper limit of normal), which is higher than what has been previously reported. For example, in a study of 996 consecutive MM patients, 11% of patients had elevated serum LDH levels^17^; similarly, in a study of 203 patients with symptomatic MM, 7% had elevated LDH.^18^ The higher rate of elevated LDH reported here may suggest shorter OS for our cohort of patients, compared with the original dataset from which the R‐ISS system was developed (where only 13% of evaluable patients had elevated LDH).^1^ In an interesting manner, in the current analysis, when high vs normal LDH was evaluated as a stand‐alone marker in a univariate analysis undertaken to explore the significance of different risk factors, it did not show any significant impact on OS.

Del(17p) is often encountered in patients with MM and is considered a predictor of adverse outcomes (ie, it has a negative impact on PFS and OS).^19^, ^20^ In the current cohort, we failed to show a significant impact of del(17p) on OS as a stand‐alone marker (HR [positive vs negative]: 1.42 [95% CI: 0.97‐2.07; *P* = .070] for OS from diagnosis; HR: 1.38 [95% CI: 0.94‐2.01; *P* = .097] for OS from treatment initiation). The fact is that prognosis of MM patients presenting with del(17p) is highly variable. It has been recently suggested that the clone size plays major role in prognosis of patients with del(17p). The patients with 10%‐60% of del(17p) were shown to have longer survival as published by Merz et al^21^ in their recent work. An et al^22^ suggested similar cutoff of 50% in their work. The interpretation and different cutoffs used might produce a bias in our results. Mutations in TP53 domain are tightly bound to del(17p) in MM as showed by Lodé et al.^23^ They demonstrated that 0% of MM patients without del(17p) presented with a mutation in TP53; conversely, not all (ie, only 37%) of patients with del(17p) exhibited a TP53 mutation. This fact may explain why only a cytogenetic test without further molecular analysis might fail to show a significant impact on patient survival. Nonetheless, the results regarding LDH, del(17p), and t(14;16) and OS (and t(14;16) and PFS) in the current analysis are inconsistent with previous research that showed chromosomal abnormalities (del(17p) and/or t(4;14) and/or t(14;16)) and LDH each had significant prognostic value in terms of survival.^1^, ^16^

### 4.2 Impact of treatment administered

Both indices of risk stratification appeared to have an effect (ie, have prognostic value) on OS in the overall study population. This finding held true for patients who received new drugs and, to some extent, patients who did not receive new drugs. Although statistical significance was not demonstrated for all comparisons in the latter group, hazard ratios suggested an increased mortality risk in higher vs lower risk patients.

Although the prognostic value of both risk stratification indices appeared to apply to patients who had not undergone ASCT, the picture was less clear in those who had undergone this form of treatment, particularly for the IMWG score; notably, we failed to show an impact of the IMWG score on OS in a transplant setting. The original work describing the value of IMWG risk stratification was based on a pooled analysis of patients treated with transplantation or conventional treatment; however, for the subgroup treated with high‐dose melphalan and ASCT, the model worked just as well.^6^ A possible explanation for the findings of the current study could be that the small number of ASCT patients, particularly within the high‐risk groups, limited the validity of the indices; indeed, only 32.6% of patients overall underwent ASCT (compared with 59% of patients in the original IMWG work), with rates of 28.7% in IMWG high‐risk patients and 24.8% in R‐ISS Stage III patients. Possible reasons for the low uptake of ASCT in the current study may include progression or death during induction chemotherapy or a suboptimal health status. In particular, poor performance status reported in our cohort of Stage II and Stage III R‐ISS patients could potentially worsen the outcome of these patients (10.6% and 14.3% PS 4) due to the fact that proper treatment could not be delivered. Performance status is an important independent prognostic indicator of survival^24^ not reflected in R‐ISS. There is also the fact that R‐ISS patient Stage II and Stage III are older than those of Stage I and poor performance status as well as more cytogenetic changes are expected in this population. The process of ASCT itself might also have influenced the prognostic value of risk stratification; that is, ASCT might partly overcome the predictive power of conventional predictors, changing a patient's prognosis vs the period prior to ASCT. No maintenance treatment is currently approved in our country; therefore, no data on possible impact of this issue are available.

### 4.3 Overall results

In the current dataset, median OS in the overall study population was 47.7 months (95% CI: 39.5‐55.9), which is somewhat shorter than what has been reported by other investigators.^1^, ^16^ Data from a study that evaluated the R‐ISS algorithm in 3,060 patients with newly diagnosed MM reported a median OS of 83 months for R‐ISS Stage II patients and 43 months for R‐ISS Stage III patients.^1^ In another study that aimed to validate the R‐ISS in an independent cohort of 475 unselected, consecutive patients with symptomatic MM treated with contemporary regimens, the estimated median OS was 63 months.^16^ Such between‐study differences in OS may be explained by several factors, including the difference in study setting. The current analysis included unselected patients in a real‐world setting, while one of the comparator studies included selected patients who were participating in experimental trials.^1^ This is relevant as data show that clinical trial participants are typically younger and healthier than the overall cancer population, resulting in differences in OS between trial participants and real‐world patients.^25^, ^26^ Indeed, the median age in the current analysis was 66 years, compared with 62 years in the comparator study undertaken in clinical trial participants.^1^ Another contributing factor to the lower OS rates in the current analysis could be the markedly lower proportion of patients who had undergone ASCT in the current study (32.6%) vs the comparator study in patients in clinical trials (60%).^1^ Numerous studies have shown that intensive therapy with ASCT is associated with improved survival, compared with conventional chemotherapy in patients with newly diagnosed MM; however, this type of treatment is typically reserved for patients who are aged 65 years or younger.^27^ In the other comparator study,^16^ which was also conducted in a real‐world setting, only 36% of patients were reported to have undergone ASCT, which is similar to the rate in the current study. In an interesting manner, OS in R‐ISS high‐risk patients in both of the real‐world studies was comparable (29 months in the earlier study vs 34.1 months in the current study); this similarity suggests a more realistic expectation of OS in a general MM population. At last, the shorter OS in the current study is likely influenced by reimbursement regulations and the corresponding availability of novel agents in the Czech Republic (eg, lenalidomide was not available for continuous treatment until 2016). Regarding OS, it is worth noting that the median OS of high‐risk (R‐ISS Stage III) patients was 34.1 months in the current analysis, compared with an estimate of less than 2 years (despite the use of novel agents) in patients who are considered high risk according to IMWG criteria.^11^ On the one hand, this increase in OS highlights the progress made in diagnosing and treating patients with MM, even those with a poor prognosis. On the other hand, it highlights the need for predictors of early relapse so that treatment can be adjusted in order to extend survival.

## 5 LIMITATIONS OF THE STUDY

The current study has an obvious limitation in that the results are based on patients in central and eastern Europe and therefore may not be generalizable to the global MM population as a whole. In addition, owing to missing data relating to genetic markers of interest, it was not possible to include all of the patients in the RMG in the current analysis; although this may introduce bias, it should be noted that the 555 patients included represents a substantial sample size, giving confidence in the conclusions drawn from the data. However, it is acknowledged that there were small sample sizes for some of the subgroup analyses (eg, the low number of patients who did not receive new drugs and who received ASCT). In an interesting manner, information about the number of patients who were ineligible to participate in the current analysis because of missing data relating to genetic markers provides a useful insight into the day‐to‐day practicality of evaluating cytogenetic abnormalities in a real‐world setting. Such challenges, however, are not restricted to clinical practice; in the previously described study that evaluated the R‐ISS algorithm, more than 30% of patients in experimental clinical trials did not have chromosomal abnormality data available (in addition to simultaneous ISS and LDH data). One of the major limitations is the heterogeneity of cutoff levels for evaluation of chromosomal abnormalities. It is important to note that this is an evolving field and the cutoffs used during the past is not valid nowadays, but the retrospective nature of data does not allow us to precisely differentiate the cutoffs. Furthermore, as with all retrospective registry‐based approaches, a central limitation is the reliance on accurate and complete patient records and data collection, and also the possibility of researcher/physician selection or information bias.

## 6 STRENGTHS OF THE STUDY

The RMG includes information from a broad range of real‐world MM patients across various categories (eg, use and nonuse of new drugs and ASCT). Owing to the noninterventional nature of the RMG, no specific drugs or treatment procedures are required for patients to be included, ensuring that patients are in a naturalistic setting where treatment choice is based on the current standard of practice and/or available treatments. Second, the registry is robust in that data are prospectively collected, regularly monitored, and validated by an external monitor. Third, the database allows an analysis of multiple baseline factors that may influence OS, including those that are not accounted for by the IMWG and R‐ISS indices such as age, comorbidities, and performance status.

## 7 CONCLUSION

Using data from the Czech Myeloma Group RMG, it was shown that the IMWG and R‐ISS risk stratification indices are applicable to patients with MM in routine clinical practice. In addition to supporting previous validation studies conducted primarily in patients participating in experimental clinical trials, the current analysis provides important information about baseline factors that may influence OS in patients with MM, including those that do not form part of the IMWG and R‐ISS indices. There is merit in conducting an analysis of outcomes in larger populations of patients who did not receive new drugs and who did receive ASCT. Nonetheless, the current analysis, along with additional analyses by other researchers, confirms the validity of risk stratification using the IMWG and R‐ISS indices in a broad range of patients with MM. Beyond simple prognostication, defining MM subgroups, as per the IMWG and R‐ISS, will prove useful in providing suitable patient counseling, delivering more effective personalized therapies, and facilitating better between‐trial comparisons of patient groups. From the current data, R‐ISS seems to show better stratification in a real‐world setting especially among patients not treated with ASCT.

## CONFLICT OF INTEREST

VM has consulted for Amgen, Bristol‐Myers Squibb, Celgene, Janssen‐Cilag, and Takeda; received grant support from The Binding Site, honoraria from Amgen, Bristol‐Myers Squibb, Celgene, and Janssen‐Cilag, and has been involved in advisory boards for Amgen, Bristol‐Myers Squibb, Celgene, Janssen‐Cilag, and Takeda. RH has consulted for Amgen, Bristol‐Myers Squibb, Celgene, Janssen‐Cilag, and Takeda; received grant support from Takeda and Janssen‐Cilag, honoraria from Amgen, Bristol‐Myers Squibb, Celgene, Janssen‐Cilag, and Takeda, and has been involved in advisory boards for Amgen, Bristol‐Myers Squibb, Celgene, Janssen‐Cilag, and Takeda. LB, AH, JJ, PJ, AJ, PK, MK, JM, PM, PP, TP, LP, JR, IS, JS, MS, LSt, LSz, and MW have no conflict of interests to declare.
